# Differential effects of jump versus running exercise on trabecular bone architecture and strength in rats

**DOI:** 10.20463/pan.2020.0001

**Published:** 2020-03-31

**Authors:** Yong-In Ju, Hak-Jin Choi, Kazuhiro Ohnaru, Teruki Sone

**Affiliations:** 1 Department of Health and Sports Sciences, Kawasaki University of Medical Welfare, Kurashiki Japan; 2 School of Sport for All, Kyungwoon University, Gumi Republic of Korea; 3 Department of Orthopedic Surgery, Kawasaki Medical School, Kurashiki Japan; 4 Department of Nuclear Medicine, Kawasaki Medical School, Kurashiki Japan

**Keywords:** different exercise types, trabecular bone microarchitecture, trabecular bone strength, finite element analysis (FEA)

## Abstract

**Purpose:**

This study compared differences in trabecular bone architecture and strength caused by jump and running exercises in rats.

**Methods:**

Ten-week-old male Wistar rats (n=45) were randomly assigned to three body weight-matched groups: a sedentary control group (CON, n=15); a treadmill running group (RUN, n=15); and a jump exercise group (JUM, n=15). Treadmill running was performed at 25 m/min without inclination, 1 h/day, 5 days/week for 8 weeks. The jump exercise protocol comprised 10 jumps/day, 5 days/week for 8 weeks, with a jump height of 40 cm. We used microcomputed tomography to assess microarchitecture, mineralization density, and fracture load as predicted by finite element analysis (FEA) at the distal femoral metaphysis.

**Results:**

Both jump and running exercises produced significantly higher trabecular bone mass, thickness, number, and fracture load compared to the sedentary control group. The jump and running exercises, however, showed different results in terms of the structural characteristics of trabecular bone. Jump exercises enhanced trabecular bone mass by thickening the trabeculae, while running exercises did so by increasing the trabecular number. FEA-estimated fracture load did not differ significantly between the exercise groups.

**Conclusion:**

This study elucidated the differential effects of jump and running exercise on trabecular bone architecture in rats. The different structural changes in the trabecular bone, however, had no significant impact on trabecular bone strength.

## INTRODUCTION

Regular exercise during childhood and adolescence, in general, is known to be a major factor affecting the achievement of peak bone mass^1,2^. That is, increased mechanical loading leads to gains in bone mass in childhood and the preservation of bone mass in adulthood. Not all types of exercise, however, have the same beneficial effects on the bone. Over the last several decades, numerous researchers have investigated the types of exercise that are more effective in improving bone mass and strength. Among the various types of exercise in animals, high-impact loading such as jumping seems to be more beneficial for increasing bone mass and strength than low-impact loading such as running^3-5^. Similar results have been shown in human experiments^6,7^. These results suggest that higher rates of strain occur with jumping activities than with running, despite similar magnitudes of strain^8,9^.

Several researchers have studied the effects of exercise cessation on bone mass in human and animal models^10-22^. Considerable disagreement remains over whether the bone mass acquired through these exercises is maintained, decreased, or lost after exercise cessation. The bone mass gained by running exercise is lost when exercise is completely ceased^13,21,22^. In contrast, other studies have suggested that bone mass gained by jump exercise is maintained after exercise cessation^12,18^. Thus, the differences in mechanical loading exerted by jump and running exercises may have different manifestations on bone adaptations. One reason for the difference in bone mass maintenance observed among the above-mentioned studies is thought to be the difference in the surface area of the trabecular bone. The turnover rate of the trabecular bone is generally accepted to be more rapid than that of the cortical bone due to the greater surface area of the trabecular bone^23^. Accordingly, the rate of bone mass reduction after exercise cessation may differ according to the bone structure, even if the amount of total cancellous bone mass remains the same. We previously reported that both jump and running exercises during the remobilization period after suspension-induced osteopenia can restore the integrity of the femoral trabecular architecture in young growing rats^24^. Interestingly, the effects on cancellous bone mass differed between jump and running exercises in that jumping increased trabecular bone volume mainly by thickening existing trabeculae, whereas running did so mainly by creating new trabeculae. Although a differential effect on trabecular architecture between these two types of exercise was observed in bone deteriorated by hindlimb unloading, it is unclear whether similar changes may occur in normal rats, and thus, we examined that in this study.

Osteoporosis is usually defined as a chronic skeletal disorder characterized by compromised bone strength that increases the risk of fracture. This definition emphasizes the importance of bone strength; therefore, understanding bone strength is vital for the reduction of fracture risk. While bone mass is a strong predictor of fracture, the ability of bones to resist fractures depends on the microarchitecture and tissue quality of trabecular bone. The role of these varying architectural foundations in bone strength, however, remains unclear. A previous study indicated that increased bone fragility and fracture risk may simply be due to a smaller trabecular number and reduced trabecular thickness^25^. So far, only two studies appear to have specifically focused on the relative effect of age-related reductions in the thickness and number of trabeculae on mechanical properties, showing that a decrease in trabecular number has a much greater negative impact on bone strength than does the loss of an equal amount of bone through trabecular thinning^26,27^. This indicates that the same increase in trabecular bone mass might be associated with different mechanical properties of trabecular bone determined by trabecular number or thickness. From these observations, we speculated that running exercise would result in a significantly higher trabecular number compared with jump exercise. In addition, we considered that a larger number of trabeculae might contribute more to trabecular bone strength than thicker trabecular bone of the same mass. No direct comparison has been made between jump and running exercises in terms of the structural characteristics that would determine the strength of the trabecular bone. This study sought to compare the effects of jump and running exercises on trabecular bone architecture and determine their significance in terms of bone strength.

## METHODS

### Animals and experimental overview

Forty-five male Wistar rats (body weight, 250–280 g) were used in this study. Animals were obtained from CLEA Japan (Osaka, Japan) at 9 weeks old. All rats were housed in individual cages in a light-controlled environment (12:12-h light/dark cycle) at a constant temperature (22 ± 1°C) and humidity (60 ± 5%). Rats were provided with a commercial standard diet (MF; Oriental Yeast Co., Chiba, Japan) that included 1.15% calcium and 0.88% phosphorus. Body weight and food intake were measured daily before exercise. The food intake for all rats was monitored with the maintenance of pair-feeding of control rats throughout the experiment. Access to water was unrestricted. All experimental procedures and animal care in this study were performed in accordance with institutional and national guidelines and regulations and were approved by the Committee for the Ethics of Animal Experiments at Kawasaki University of Medical Welfare (Permit Number: 10-011). Rats were euthanized with a single intraperitoneal injection of sodium pentobarbital after the 8-week experiment. All possible efforts were made to minimize suffering. Rats were habituated to the diet and new environment for 1 week. The health and behavior of animals were monitored at least twice daily. After 7 days of acclimation, rats were randomly assigned into three groups as follows: a sedentary control group (CON, n=15), a treadmill running group (RUN, n=15), and a jump exercise group (JUM, n=15). Soon after euthanasia, the right calf muscles of each rat were collected and immediately weighed. The right femur was excised from each rat and cleaned of soft tissue. Femoral length was measured using digital calipers. The femurs were stored at -40 °C until needed for further measurements.

### Exercise conditions

Jump exercise conditions were determined according to our previous publications^24,28^. The jump exercise program was performed 10 times/day, 5 days/week for 8 weeks. Rats in the jump exercise group were individually placed at the bottom of a special wooden box surrounded with boards. The box height was adjustable. Rats were to jump, grasp the top of the board with the forelimbs, and climb up the board. They were then returned to the floor of the cage to repeat the procedure. The rats were initially forced to jump by an electric stimulus, but as they became accustomed to the exercise, the electric stimulus was used less frequently. The box height was initially 25 cm and was progressively increased to 40 cm during the first week.

The treadmill running exercise protocol was implemented according to our previous publications^24,29^. Rats in the running exercise group had a running program on a motor-driven treadmill (KN. 73 Tread-Mill RM. 5; Natume, Tokyo, Japan) for 8 weeks. The treadmill speed and the duration of each running session were progressively increased from 10 m/min for 10 min to 18 m/min for 50 min during the first week and to a final level of 25 m/min without inclination for 60 min within the following week. Rats were maintained at this final speed and duration for the remainder of the training program. The rats were stimulated to run with compressed air blown from behind in lieu of electrical stimulation. The front half of the treadmill was covered in black paper to keep the area dark, as rats are more active in darkness. The intensity of exercise during the experiment was considered moderate-intensity (about 50-60% of maximum oxygen consumption; VO_2max_), according to measurements from our previous study^29^.

### Measurement of 3D architectural indices and mineralization density of cancellous bone

**Figure 1.**
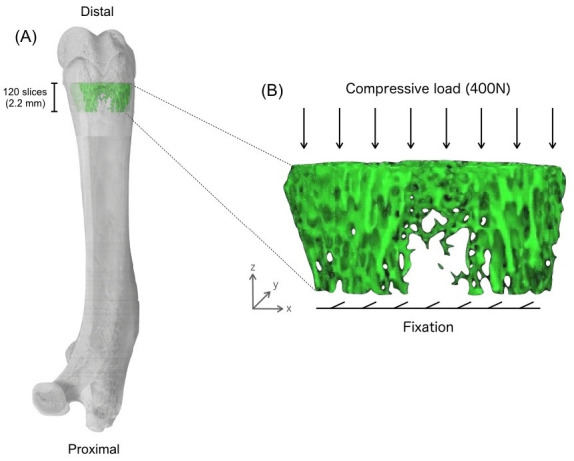
Location of the volume of interest in the distal femoral metaphysis for micro-CT analysis. (A) Selected region of the distal metaphysis of the femur for calculating trabecular bone parameters. (B) FEA for predicting trabecular bone strength. Compression test in the axial direction with a 400-N load applied to the distal surface of the femur while displacement in all other directions of the proximal surface is fully constrained.

Bone microarchitecture in the right femur was evaluated using microcomputed tomography (micro-CT) (Ele Scan mini; Nittetsu Elex, Tokyo, Japan). This apparatus is based on fan-beam tomography and is able to function in multislice mode. An X-ray tube with a microfocus (spot size of 6 × 8 μm) and a maximum resolution of 4 μm (in pixel size) was used. Parameters selected for this study included a source energy of 45 kVp and 90 mA to obtain optimal contrast between bone and soft tissue. A 0.1-mm copper plate served as the X-ray filter. The sample area selected for scanning was positioned at a distance of 2.8-3.0 mm proximal from the distal femoral end, including the border between the distal metaphysis and growth plate. The distal femur has a larger volume of cancellous bone available for 3D analysis and was, thus, selected over the proximal femur for analysis. A total of 300 consecutive tomographic slices with a slice thickness of 18.1 μm (approximately 5.4 mm) were acquired. Digital data were reconstructed to obtain CT images with a pixel size of 18.1 μm in 512 × 512 matrices. After micro-CT, the original image data were transferred to a workstation and structural indices were calculated using 3D image analysis software (TRI/3D-BON; Ratoc System Engineering, Tokyo, Japan). The images of interest were defined as the 120 slices above the most proximal portion of growth plate (Fig. 1A). The resulting gray-scale images were segmented using a 3 × 3 median filter to remove noise with a fixed threshold of 120 (0-255 range) to extract the mineralized bone phase. Isolated small particles in the marrow space and isolated small holes in bone were removed using a cluster-labeling algorithm. Cortical and trabecular bone were subsequently separated and structural indices were calculated. Bone surface area (BS) and bone volume (BV) were calculated using a tetrahedron meshing technique generated using the marching cubes method. Total tissue volume (TV) was calculated as the volume of the entire scanned sample. Trabecular bone volume fraction (BV/TV) was then calculated from BV and TV. Trabecular thickness (Tb.Th), trabecular number (Tb.N), and trabecular separation (Tb.Sp) were calculated by measuring 3D distances directly in the trabecular network^30^. Connectivity density (β1/TV)^31^, trabecular bone pattern factor (TBPf)^32^, and structural model index (SMI)^33^ were calculated directly from segmented voxel representations. Total BV was measured as a volume of all cortical and cancellous bone, including marrow space, in the region of interest for analyzing trabecular bone architecture. In addition, the average degree of trabecular bone mineralization density (mg/cm^3^) was calibrated to the manufacturer’s hydroxyapatite phantom (6 × 1 mm; 200 to 800 mg/cm3; Kyoto Kagaku, Kyoto, Japan). Analysis was performed using TRI/3D-Bon BMD software (TRI/3D-BON; Ratoc System Engineering, Tokyo, Japan) and the average degree of mineralization was measured in the region of interest. BMD phantoms were assessed by CT under the same conditions as actual bone.

### Finite Element Analysis (FEA) for micro-CT images

All FEA were performed using TRI/3D-FEM64 FEA software (RATOC System Engineering). Reconstructed 3D grayscale images of the distal femur obtained by micro-CT (120 slices, pixel size 18.1 μm) were used for FEA (Fig. 1B). The sample model was loaded by a distributed force applied perpendicularly to the distal surface of the femur while displacement in all other directions of the proximal surface was fully constrained. The magnitude of the applied force was chosen to be 400 N. The fracture load was determined as that level inducing fractures in 2.8% of all voxels. Young’s modulus of the trabecular bone was calculated from the CT number using the formula described by Carter and Hayes^34^. For all materials, a Poisson’s ratio of 0.3 was specified.

### Statistical analysis

All statistical analyses were carried out using IBM SPSS Statistics version 19.0 software package (IBM, Armonk, NY). Comparisons among categories were statistically processed by one-way analysis of variance (ANOVA) followed by Tukey’s post hoc analysis. Data were tested for normal distribution using the Shapiro-Wilk test and homogeneity of variance using Levene’s test before ANOVA analysis. If data failed these tests, the significance of differences across groups was evaluated using the Kruskal-Wallis nonparametric test. All data are expressed as means ± SD. The level of statistical significance was set at P<0.05 for all analyses.

## RESULTS

### Body weight, calf muscle weight, and femoral length

Body weight before and after the experiment, calf muscle weight, and femoral length of rats from each group are shown in Table 1. During the experiment, no differences in body weight were noted between the sedentary control rats and the rats from the two exercise groups. Calf muscle weight was similar among the sedentary control group and the two exercise groups at the end of the experiment. Femoral length was also similar among the three groups. Jump and running exercises did not affect either calf muscle weight or femoral length.

**Table 1.** Body weight, calf muscle weight, and femoral length of experimental rats

	CON (n=15)	JUM (n=15)	RUN (n=15)
Body weight before experiment (g)	320.7 ± 10.55	321.5 ± 09.18	313.7 ± 11.99
Body weight after experiment (g)	398.9 ± 14.14	398.4 ± 15.39	386.5 ± 25.99
Calf muscle weight (g)	2.40 ± 0.14	2.56 ± 0.51	2.39 ± 0.20
Femoral length (mm)	36.39 ± 1.51	36.29 ± 1.13	35.87 ± 1.52

All values represent mean ± SD. n, number of rats in each group; CON, sedentary control group; RUN, treadmill running exercise group; JUM, jump exercise group.

### Trabecular microarchitecture, mineralization and FEA-estimated fracture load

**Figure 2.**
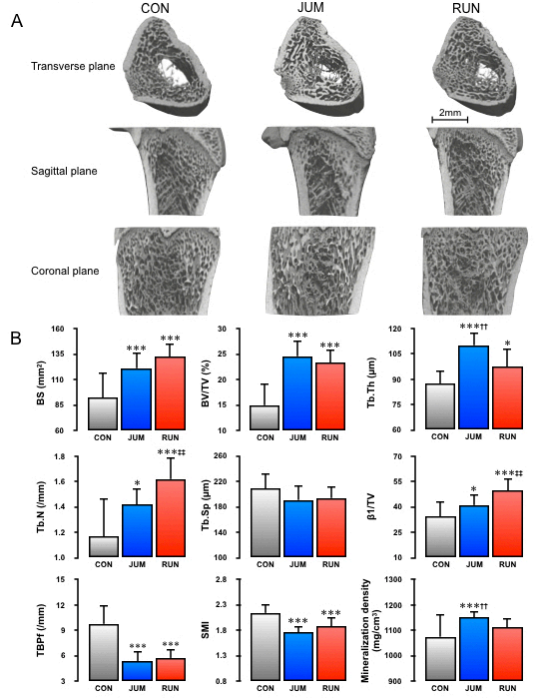
Micro-CT analysis of the trabecular bone architecture of the distal femoral metaphysis in rats. (A) Representative three-dimensional (3D) reconstructed images of each group are shown (transverse sectional, top; sagittal, middle; coronal, bottom). CON, sedentary control group; RUN, treadmill running exercise group; JUM, jump exercise group. (B) Trabecular microarchitecture parameters of the distal femoral metaphysis are shown as: BS, bone surface; BV/TV, trabecular bone volume fraction; Tb.Th, trabecular thickness; Tb.N, trabecular number; Tb.Sp, trabecular separation; β1/TV, connectivity density; TBPf, trabecular bone pattern factor; SMI, structure model index; degree of trabecular bone mineralization. All values represent means ± SD. Significant difference vs. CON group: **p<0.01; ***p<0.001. Significant difference vs. JUM group: ^‡‡^p<0.01. Significant difference vs. RUN group: ^††^p<0.01.

**Figure 3.**
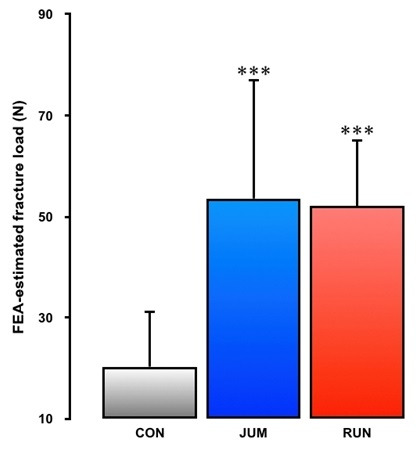
Fracture load as predicted by FEA. All values represent means ± SD. CON, sedentary control group; RUN, treadmill running exercise group; JUM, jump exercise group. Significant difference vs. CON group: ***p<0.001.

Results for 3D microstructural parameters and mineralization in the distal metaphysis of the femur are shown in Figure 2B. In terms of femoral trabecular bone parameters, BS, BV/TV, Tb.Th, Tb.N, and β1/TV in both RUN (43%, p<0.001; 56%, p<0.001; 11%, p<0.05; 38%, p<0.001; 45%, p<0.001, respectively) and JUM groups (31%, p<0.001; 64%, p<0.001; 26%, p<0.001; 22%, p<0.05; 20%, p<0.05, respectively) were significantly higher than in the CON group. In contrast, TBPf and SMI were significantly lower in the RUN (-41%, p<0.001; -12%, p<0.001, respectively) and JUM groups (-45%, p<0.001; -18%, p<0.001, respectively) than in the CON group. A trend was evident toward lower Tb.Sp in the RUN and JUM groups than in the CON group, although the difference was not significant. Compared with the RUN group, Tb.Th increased 13% in the JUM group (p<0.01). On the other hand, Tb.N and β1/TV increased by 12% and 17% in the RUN group than in the JUM group (p<0.01 and p<0.01, respectively). Total bone volume did not differ significantly among the three groups (CON: 57.94 ± 8.03, RUN: 59.05 ± 8.40, JUM: 60.82 ± 8.57, respectively). The degree of trabecular bone mineralization was significantly higher in the JUM group than in the CON and RUN groups (7%, p<0.001 and 4%, P<0.01, respectively). Figure 2A shows the typical trabecular microarchitecture in the distal femoral metaphysis for a rat from each group. Micro-CT images from both jumping and running rats demonstrate diffuse increases in trabeculae. Furthermore, differential effects of jump versus running exercises on trabecular microarchitecture can be confirmed visually. No difference, however, was found in bone mineralization between the two exercise groups. In addition, FEA-estimated fracture loads in both RUN (p<0.001) and JUM (p<0.001) groups were significantly higher than in the CON group, but no significant differences were observed between the two exercise groups (Fig. 3).

## DISCUSSION

Our primary focus in this study was investigating the relationship between different exercise types and their effects on trabecular bone architecture and strength. The main findings were that the effects of jump exercise on trabecular bone mass and strength of the distal femur were primarily achieved by alterations in trabecular thickness, whereas running exercise induced the same effects by enhancing trabecular number. On the other hand, the extent of the increase in bone strength was similar between these two types of trabecular alterations.

Several studies have previously found that the increase in trabecular bone mass by jump exercise is primarily due to increased trabecular thickness rather than noticeable changes in trabecular number^3,35^, whereas treadmill running exercise induced the same effects by increasing trabecular number^29,36^. This suggests that jump and running exercise conditions might exert different influences on the structural characteristics of trabecular bone. No comparative studies, however, have broadly examined the architectural changes in the trabecular bone of rats after jump and running exercises. In the present study, both jump and running exercises induced 22% and 38% increases in trabecular number, respectively, and 26% and 11% increases, respectively, in trabecular thickness in the distal femoral metaphysis compared to the sedentary control group. Jump and running exercises caused different changes in trabecular bone architecture, as found in our previous study^24^. Trabecular number in the running exercise group was 12% higher compared to that of the jump exercise group, while trabecular thickness in the jump exercise group was 13% higher than that of the running exercise group. This hypothesis is circumstantially supported by the observation that bone surface area tended to be lower in the jump exercise group than in the running exercise group, although the difference was not statistically significant. Collectively, these results suggest that jump and running exercises have different mechanisms of action (strain rate, strain magnitude, cycle number, loading direction, etc.) on the structural characteristics of trabecular bone for reasons that remain unclear.

High bone density is directly related to bone strength, but density alone cannot explain the variability in the mechanical behavior of trabecular bone. Other than bone mass, bony microarchitecture is also believed to play an important role in determining the mechanical properties of the trabecular bone. This study found a significantly larger trabecular number in treadmill running-exercised rats, while jumping-exercised rats tended to show increases in trabecular thickness. Since an increase in trabecular connectivity or decrease in trabecular bone pattern factor is usually coupled with increases in bone strength^37^, it is conceivable that changes in trabecular bone pattern factor and connectivity density contributed to the beneficial effects of jump and running exercises on the mechanical strength of the trabecular bone. On the other hand, connectivity density was significantly higher with running exercise than with jump exercise. A study implementing a mechanical stimulation in ovine femora reported that ultimate strength correlated most highly with a structural model index, followed by bone volume fraction^38^. In the present study, jump and running exercises significantly decreased the structural model index compared to the sedentary control group, suggesting a concomitant increase in mechanical strength. Jump and running exercises both increased the fracture load predicted by FEA based on micro-CT of the distal femoral metaphysis and did not result in any clear difference in fracture load along the axial direction. These results did not support the hypothesis that a higher trabecular number plays a much more important role in determining trabecular bone strength than thicker trabecular bone of the same bone mass. The reason for the difference between the present findings and the two above-mentioned studies is unclear at this point. Studies with larger sample sizes are needed to confirm some of our study findings.

Some methodological limitations must be taken into consideration before concluding this discussion. First, the loading condition used in the FEA was streamlined. We simulated a compression test on a planar parallel slice of the distal femoral metaphysis, which might not provide an accurate representation of the loading applied to this region during running and jump exercises. Further detailed data under different loading conditions (e.g., load applied in the diagonal direction) will be helpful to validate differential effects among different types of mechanical loading on structural bone architecture. Additionally, we did not measure the breaking force in cancellous bone per se at the distal femoral metaphysis. This information may be very helpful for validating estimated fracture load derived by the FEA model. Second, this study did not include a direct comparison of trabecular microstructural indices with dynamic parameters such as bone formation and resorption derived from histomorphometric analyses or measurement of serum markers of bone turnover. We cannot confirm the tissue-level or systemic mechanisms underlying the different functional adaptations of trabecular architecture to mechanical loading observed in the present study. Further detailed dynamic histomorphometric measures and serum bone marker assays may prove helpful in validating the mechanisms behind the differential effects among different types of mechanical loading on structural bone architecture. Finally, we did not measure the intensity of jump and running exercises during experiments. Judex and Zernicke^9^ compared the mechanical loading characteristics produced by running and drop jumps and found that drop jumping involved much larger maximal strain rates than running protocols in growing roosters. Equalizing loading conditions among different modes of exercise is difficult. In the present study, however, exercise protocols were designed based on our previous study^24^ so that comparable increments in trabecular bone mass could be expected from both exercises. As expected, trabecular bone volume and mineralization were similar in jump and running exercised rats. Some studies have reported the relationship between exercise intensity and bone mass^39-42^. It is reported that high-intensity exercise (80% of VO_2max_) reduces longitudinal bone growth and increases bone loss in rats^40,42^, while low intensity (40% of VO_2max_) showed a decreased trabecular bone volume of the femur^39^. Accordingly, it is well established that exercise intensities below 40% VO_2max_ and above 80% VO_2max_ do not improve bone loss in rats^41^. Therefore, the present results might qualitatively differ if more intense exercise protocols were applied.

In conclusion, we have demonstrated that while both jump and running exercises enhanced trabecular bone mass and strength in rats, they exerted different effects on trabecular microarchitecture in the distal femoral metaphysis. Running exercise predominantly enhanced trabecular number, while jump exercise predominantly enhanced trabecular thickness. The FEA-estimated fracture load of the distal femoral metaphysis showed no significant difference between the two exercises. These results suggest that both jump and running exercises promote gains in the trabecular bone mass and strength of the distal femoral metaphysis through different architectural methods in rats, but these different aspects of the trabecular microarchitecture have no significant effects on trabecular bone strength.

